# Underestimation of prevalence of raised blood sugar from history compared to biochemical estimation: support for the WHO rule of halves in a population based survey in Eritrea of 2009

**DOI:** 10.1186/s40064-015-1516-3

**Published:** 2015-11-24

**Authors:** Jacob Mufunda, Yohannes Ghebrat, Abdulmumini Usman, Goitom Mebrahtu, Asmera Gebreslassie

**Affiliations:** World Health Oragnization Zambia Office, Lusaka, Zambia; World Health Organization Eritrea Office, Asmara, Eritrea; Ministry of Health, Asmara, Eritrea

## Abstract

To ascertain the prevalence of diabetes mellitus from history and biochemical estimation so as to attest the WHO Rule of halves in a lean population. A population based national survey on diabetes mellitus was carried out in 2009. History and fingerpicks blood analysis were examined according to recommended procedures of the WHO STEPwise approach and the WHO recommended automated machine to compare the two modalities of estimating diabetes prevalence. Over 6000 people with a response rate of 95 % and a prevalence of raised blood glucose of 5.0 %. The prevalence from history of raised blood sugar was 2.2 %. Less than half (47 %) of the persons with high blood glucose were aware of their status with less than half on treatment. Of those on treatment less than half (30 %) were well controlled. Prevalence of raised fasting blood glucose was more than double that estimated from history, with less than half of the people aware of their status and of those on treatment nearly half are under good control. The underestimation of the disease through history supports the WHO rule of halves and calls for the use of biochemical tests when estimating prevalence of diabetes in the general population or at least doubling the rate from history alone.

## Background

Diabetes mellitus, which was insignificant in its prevalence in early 1900 (Cook 1901) and was further estimated to be insignificant till the 1960s, is rapidly increasing. The global diabetes disease burden increased from 153 million in 1980 to 347 million in 2008. If the trend continues, by 2030, the projected number of people with diabetes will be 552 million which is nearly 1 in 10 adults (Unwin et al. 2011).

In 2010, 12.1 million people were estimated to be living with diabetes in Sub-Saharan Africa and this was extrapolated to increase to 23.9 million by 2030 (Sicre et al. 2009). The prevalence rates range from 2.5–8 % in the rural and urban communities of West Africa and 1–12 % in the rural and urban communities in East Africa (Mohan et al. 2013). Various studies on trends of diabetes mellitus in the African region demonstrate that there is a dramatic increase in the prevalence affecting both rural and urban communities as well as men and women equally, as in Cameroun whereby type 2 diabetes increased tenfold between 1994 and 2003 (Hanley 2007; Mufunda et al. 2006; Hall et al. 2011; Usman et al. 2006).

The increase in diabetes is proposed to be the result of the demographic change (increase in older age group), urbanization, and associated changes in risk factors such as alcohol use, tobacco smoking, obesity and physical inactivity (Kaufman et al. 1999).

The raised fasting blood glucose (RFBG) level and the coexistence with other non-communicable diseases and related syndromes predispose one to the vital organ damage (Codreanu et al. 2012). It is estimated that 50 % of people with diabetes die of cardiovascular disease; heart disease and stroke being the most common whereas about 2 % of people become blind and roughly 10 % develop severe visual impairment after 15 years of living with uncontrolled diabetes. Among people with diabetes the overall risk of dying is at least double the risk of their peers without diabetes (Mills 2015). The African Region has the highest proportion of undiagnosed diabetes mellitus (78 %). (Unwin et al. 2011).

The rule of halves was first proposed for non-insulin dependent diabetes in the USA in 1947 (Wilkerson and Krall 1947) and confirmed in the UK in 1964 (Sharp et al. 1964). The rule states that approximately half of most common chronic disorder is not detected, and half of those detected are not treated, and that half of those treated are not controlled. The rule of halves still holds true for diabetes (Hayes and Harries 1984) and other chronic diseases validating (Smith et al. 1990; Speight et al. 1983) the WHO rule of halves. Based on these previous studies, it was assumed that the rule of halves could provide a good extrapolation on the actual estimates of the prevalence of diabetes (Hayes and Harries 1984) and related organ damage as gathered from history and being on medication.

The aim of this study was to provide a more accurate estimate of high blood sugar and comparing with estimates from history of the disease and validate the rule of halves for diabetes prevalence in Eritrea.

## Methods

### Study population

A cross sectional population based survey using the WHO STEP 3 method was conducted to determine the prevalence of the common risk factors of the major NCDs was conducted in 2009 in all the six administrative Zobas (Regions) of Eritrea. Persons in the age group 25–74 years participated.

Multistage cluster sampling was applied with the guide of the WHO Stepwise approach for the NCD risk factors surveillance, adapted to the Eritrean context to reach the sample size of 6400. In the first stage from the sampling frame of 2624 clusters/villages, 162 clusters were selected by applying probability proportional to size sampling method. In the second stage 40 households were selected from each of the selected 162 clusters using linear systematic random sampling.

### Data collection

Data was collected using Personal Digital Assistants (PDAs) Hewlett Packard IPAQ model no. 110. Once the questionnaire was adapted it was tested before the actual data collection process, finally the questionnaire was uploaded into PDAs to be used by the data collectors as e-questionnaire.

### Blood sample collection and biochemical analysis

Blood samples were collected from the eligible participants in accordance with the recommended procedures of the WHO STEPwise approach manual. Prior to the collection of the blood samples participants were requested to fill in an informed consent form. Those participants who consented to participate in STEP 3 were requested to fast for 12 h prior to the blood sample collection. The blood samples were collected fingertips of the consented participants using Accutrend glucose between 0700 and 1000 h, following the overnight fast. The samples were analyzed in the field using the WHO recommended automated machine called Accutrend GCT (Accutrend GCT; Roche Diagnostics GmbH D-66298, Mannheim, Germany) and the results were expressed in mg/dl.Diabetes was classified as: Plasma venous value ≥ 7 mmol/l (equivalent to ≥ 126 mg/dl) or Capillary whole blood value ≥ 6.1 mmol/l (equivalent to ≥ 110 mg/dl).

### Data quality assurance

The data collectors and supervisors were recruited from the College of Health Sciences and Ministry of Health which are experienced in data collection and research, physical measurements and collection of blood.

In order to ensure quality and reproducibility of the survey result, clearly defined standard STEPS survey procedures were observed. In addition, random checks by field supervisors, survey coordinator and re-interviewing the important questions from at least 5 % of the respondents by different interviewers were done.

### Ethical issue

Ethical permission was obtained from the Ministry of Health, Eritrea Ethics Committee prior to the commencement of the survey. All participants signed informed consent before enrolment.

## Results

The overall prevalence of raised fasting blood glucose was 5.0 % (95 % CI 4.0–6.0 %), and was significantly higher in males (8.3 %) than in females, 4.3 % (Table 1). Tigrigna ethnic group showed the highest prevalence of raised fasting blood glucose (5.5 %), followed by Tigre (2.3 %) while the remaining seven ethnic groups combined was slightly higher (2.8 %) than that of Tigre ethnic group alone (Table 2).

**Table 1:** Prevalence of raised fasting blood glucose (RFBG) by gender

Variable	N	Prevalence (%)	Confidence intervals at 95 %
Sex			
Male	1725	8.3	6.4–10.2
Females	4540	4.3	3.4–5.2
Both sexes	6265	5.0	4.0–6.0

**Table 2:** Prevalence of raised fasting blood glucose by ethnic group

Variable	N	Prevalence (%)	Confidence intervals at 95 %
Ethnic group			
Tigrigna	3604	5.5	4.8–6.3
Tigre	1283	2.3	1.6–3.4
Other	1378	2.8	2.0–3.8

**Fig. 1:**
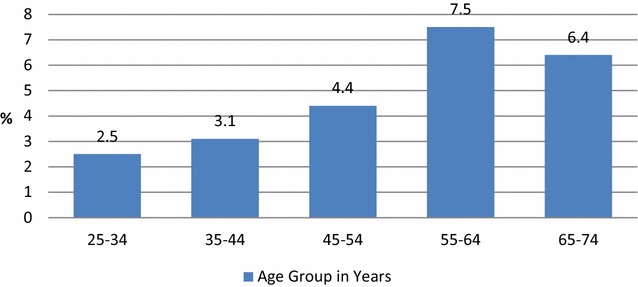
Prevalence of diabetes by age group

The prevalence of raised fasting blood glucose level increased with age being highest in the age group 55–64 years and the lowest was in the age group 25–34 years. There was a slight decrease of prevalence among the highest age group i.e. 65–74 years (Fig. 1).

Similarly, among the six regions in the country, matching the degree of urbanization; Maekel had the highest prevalence of raised fasting blood glucose (10.2 %) followed by Debub (5.1 %) and Southern Red Sea Zones (3.9 %).

As presented in Table (3), only 2.2 % of the sample had history of raised fasting blood glucose which is less than half of those with raised fasting blood sugar (5.0 %) biochemically. Among those with history of the disease, less than half (16.1 %) were on treatment and less than half (30.0 %) of those on treatment were controlled (Table 3).

**Table 3:** Knowledge, treatment and control status on RFBG

	N	%	Confidence intervals at 95 %
Knowledge status on RFBG by history (told to have diabetes)			
Raised blood sugar status in the total sample			
Raised blood sugar status known (raised)	124	2.20	1.8–2.6
Raised blood sugar status not known or not raised	5605	97.80	97.4–98.2
Raised fasting blood glucose during the survey			
Raised fasting blood glucose at day of the survey			
RFBG among those who knew their status	69	26.80	21.5–32.7
RFBG among those who did not know	188	73.20	67.5–78.5
Control status of blood sugar among diabetics			
Control status of blood sugar among those known to have diabetes			
Blood glucose controlled	55	44.4	35.4–53.5
Blood glucose not controlled	69	55.6	46.5–64.6
Proportion on treatment			
Among those who knew their status			
Raised blood sugar statues known and on treatment	20	16.10	10.1–23.8
Raised blood sugar Status known but not on treatment	104	83.90	76.2–89.9
Control status of blood glucose level			
Among those who were on treatment			
Raised blood sugar controlled	6	30.00	11.9–54.3
Raised blood sugar but NOT controlled	14	70.00	45.7–88.1

## Discussion

This cross sectional study conducted in Eritrea used the WHO STEPwise tool to determine the prevalence of raised blood sugar confirmed that less than half of the sample knew of their diabetes status (diagnosed), less than half of those diagnosed were on treatment and of those on treatment less than half had their disease well controlled. These findings validate the Rule of halves that was demonstrated in other studies. These findings corroborate findings from other studies like that from the Indian one whereby of the total estimated 16 % of diabetics nationwide, 33 % were diagnosed to have diabetes whereas double (68 %) were undiagnosed for the disease (Ranjit et al. 2011).

To determine the exact burden of NCDs and their risk factors in a country, the golden rule is to conduct a population based biochemical study. However, these studies are usually very expensive resulting in absence of adequate data for action. The lack of adequate data and underestimation of these diseases in low and middle income countries necessitates for the need of assessing a cheaper yet effective system or rule (Hart 1992). Rules, such as the WHO rule of halves, can assist a country to project towards the actual prevalence estimates of diseases at reasonable costs that in turn can be translated into appropriate control strategies. (Hart 1992).

Globally, the prevalence of raised blood sugar is generally higher among the males than the females (Mohan et al. 2013), this is not a homogenous finding. Our study had also revealed that the prevalence of raised fasting blood glucose was almost double in males compared to females. This could possibly be related to the higher prevalence of NCD risk factors in males such as high tobacco and alcohol use, low fruit and vegetables consumption among the (World Health Organization 2010; Donnelly et al. 1997).

Behavioral risk factors such as smoking could increase the risk of impaired fasting glucose by decreasing insulin sensitivity (Hilawe et al. 2013; Nakanishi et al. 2000) and in the same token men generally tend to have lower hepatic sensitivity to insulin (Færch et al. 2010) thus resulting in raised fasting blood glucose. Similarly, other studies have shown that unhealthy diet (Faerch et al. 1905) and physical inactivity (Assah et al. 1901) are associated with impaired glucose tolerance although physical inactivity was not pronounced in our survey.

Similar results were reported, one from Tanzanian where the prevalence was higher among males than the females (McLarty et al. 1989). Yet, the trend has not been consistent with conflicting reports from several other African countries, including Mauritius, Seychelles, South Africa and Zimbabwe the trend of rising fasting blood glucose level is higher in females than in males (Mufunda et al. 2006).

There are nine Ethnic groups in Eritrea. The prevalence of fasting raised blood sugar was more than double in Tigrigna ethnic group compared to Tigre and the others. This could mainly be due to genetic contributing factors as well as the high urbanization level among the Tigrigna tribe that mainly live in the Maekel region where Asmara, the capital city is located. Urbanization is observed to influence the living style including the dietary habit, physical exercise, alcohol and tobacco consumption as well as other NCD risk factors constituting a significant factor to the rise of diabetes as witnessed from other studies conducted in Cameroun (Echouffo-Tcheugui and Kengne 2011) and Durban, South Africa (Levitt et al. 1993).

Ethnicity influencing the propensity for chronic diseases diabetes included especially in a multi-ethnic population. For example non-white ethnicities were associated with higher risk than whites for developing diabetes with South Asians having the highest risk (Rosellaa et al. 2012). Yet, similar high prevalence of diabetes mellitus was observed in Cameroon whereby exposure to urban environment was strongly correlated to raised fasting blood glucose level (Sobngwi et al. 2004). Thus, considering the role of ethnicity and its risk factors, further studies are encouraged to clearly define the predisposition in Eritrea.

In conclusion, it is important to do biochemical tests when conducting estimations of diabetes in a population. However, using the rule of halves, the scarce information that is gathered from various other sources including from history and medication, can be extrapolated to provide reliable proxy estimates to the prevalence rates of chronic diseases. This eventually, could enable decision makers in low resource settings to develop appropriate and effective strategies for the control of diabetes and other NCDs in order to mitigate timely any complications that may arise from damage to the vital organs.
